# Genitourinary Tuberculosis: A Rare Cause of Obstructive Uropathy in Pregnancy

**DOI:** 10.1155/2014/985682

**Published:** 2014-06-22

**Authors:** Emily H. Adhikari, Elaine L. Duryea, Martha W. F. Rac, Jeanne S. Sheffield

**Affiliations:** Department of Obstetrics and Gynecology, University of Texas Southwestern Medical Center, 5323 Harry Hines Boulevard, Dallas, TX 75390-9032, USA

## Abstract

*Background*. A rare but morbid form of extrapulmonary tuberculosis (TB), genitourinary TB is an important cause of obstructive uropathy and is likely underdiagnosed in pregnancy. *Case*. A 30-year-old primigravida undergoing treatment for active pulmonary TB presented with anuria at 13-14-weeks gestation. Bilateral ureteral strictures above the level of the ureterovesicular junctions were seen on imaging studies. Given her pulmonary disease, her obstructive uropathy was attributed to genitourinary TB. Bilateral percutaneous nephrostomy tubes were placed during pregnancy with successful ureteral reimplantation postpartum. *Conclusion*. Genitourinary TB should be considered as an etiology of urinary tract pathology during pregnancy, especially in foreign-born and immunocompromised persons. Early recognition resulting in prompt treatment can prevent further deterioration of maternal renal function and optimize pregnancy outcomes.

## 1. Introduction

Despite a declining incidence of tuberculosis (TB) since the early 20th century, TB remains a significant global health concern. In 2011, more than 8.7 million new cases were reported worldwide [1]. Although only 9,945 new cases (3.2 cases per 100,000 persons) were reported in the United States for 2012, the percentage of TB cases among foreign-born persons has increased to a case-rate 11 times higher than in US-born persons [1]. Practitioners serving predominantly immigrant communities are more frequently faced with diagnosing and treating TB. Although the most common site of TB infection is pulmonary, extrapulmonary manifestations of TB occurred in 2,100 cases during 2012, most commonly in lymphatics, the pleural space, and bone [1]. Genitourinary occurrence of TB is rare, occurring in only 5% of extrapulmonary cases reported in the US during 2012 [1]. Considered a severe form of extrapulmonary TB, genitourinary TB can involve the retroperitoneum, adrenal gland, kidney, urinary collecting system, and reproductive organs [2]. The delay in presentation with genitourinary TB can be over 20 years after the primary TB infection [3], mandating a high index of suspicion on the part of the practitioner.

Pregnancy presents a unique opportunity for diagnosis and treatment of all forms of TB in a group of high-risk patients who may otherwise lack access to long-term medical care. We present a case of complete obstructive uropathy and acute renal failure from genitourinary TB in a pregnant woman receiving treatment for active pulmonary tuberculosis.

## 2. Case

A 30-year-old Hispanic primigravida presented to the emergency room with worsening abdominal pain, urinary retention for 4 days, and a positive urine pregnancy test. She reported a two-year history of unexplained infertility and was unaware of her pregnancy prior to presentation. Ultrasound revealed a 13-14 week intrauterine pregnancy with cardiac motion. Upon further questioning she reported a history of active pulmonary TB infection and had received 4 months of treatment with isoniazid, ethambutol, and rifampin (RIE) by the local health department. Records received from the health department revealed a sputum culture positive for* M. tuberculosis* and* M. bovis,* both resistant to pyrazinamide. She had no other medical or surgical history and denied recent travel, drug use, or homelessness. Vital signs on admission were unremarkable. Her abdomen was soft with discomfort in bilateral lower quadrants; no peritoneal signs or costovertebral angle tenderness was present. Pelvic exam revealed a mobile, anteverted, 14-week-size uterus without cervical or adnexal masses. Rectovaginal exam failed to demonstrate abnormalities of the rectovaginal septum or pelvic sidewall. A urethral catheter was placed without return of urine. Laboratory studies were remarkable for a serum creatinine of 7.5 mg/dL, serum potassium of 5.0 mmol/L, and a mild metabolic acidosis. Severe bilateral hydronephrosis and hydroureter measuring 2.6 cm on the right and 1.2 cm on the left were seen on renal ultrasound.

**Figure 1:**
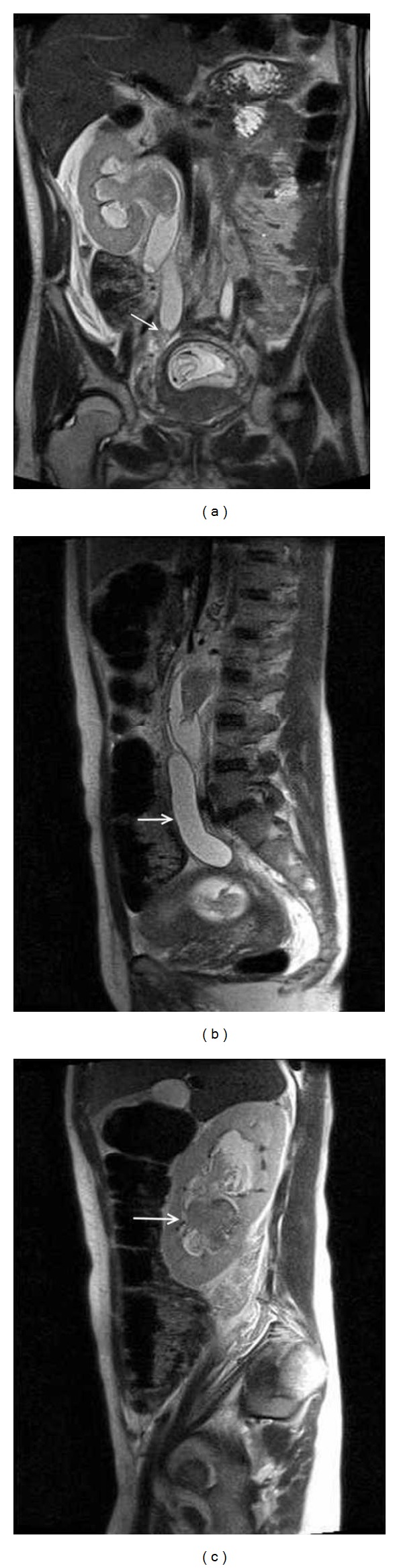
(a) MRI (coronal view) demonstrating high-grade stenosis of right distal ureter (arrow). Gravid uterus is also seen. (b) Sagittal view of tortuous left hydroureter (arrow). (c) Sagittal view of left hydronephrosis with blood (arrow) in the collecting system.

**Figure 2:**
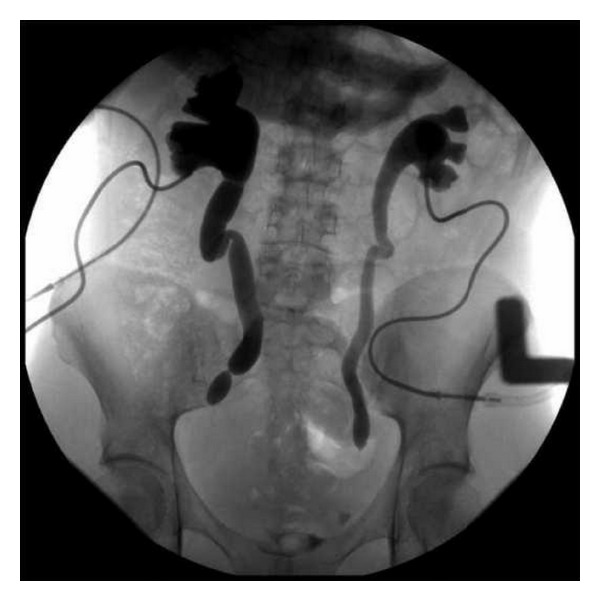
Antegrade pyelogram demonstrating multiple ureteral strictures and absence of contrast in bladder. Percutaneous nephrostomy tubes are present.

The urology service was consulted and emergent bilateral percutaneous nephrostomy tubes were placed with subsequent rapid resolution of her laboratory abnormalities. To better characterize the degree of obstruction, an MRI was obtained and revealed severe bilateral hydroureteronephrosis with high-grade strictures located 5 cm and 4.5 cm proximal to the right and left ureterovesicular junctions, respectively (Figure 1). Both ovaries appeared normal, and there were no abdominopelvic masses identified. Given the clinical picture and her ongoing treatment for pulmonary infection, as well as negative work-up for an alternative etiology thus far, genitourinary TB was suspected. Serial acid-fast bacilli (AFB) urine cultures and PCR were sent on three consecutive early morning urine specimens but were negative on follow-up. However, given the patient's partially treated pulmonary TB and the exclusion of other possible causes, the ureteral strictures were thought to be sequelae of prior genitourinary TB rather than active disease. The patient improved and was discharged home on hospital day eight. She continued her RIE therapy until completion and returned for regular prenatal visits and routine percutaneous nephrostomy tube exchanges. At 37-38 weeks gestation she underwent induction of labor for severe preeclampsia, delivering a vigorous term infant. Her postpartum course was complicated by pyelonephritis which was treated with antibiotic therapy based on microbial susceptibilities. Two months postpartum, antegrade pyelogram revealed continued complete obstruction of bilateral ureters, with stenotic segments measuring 6 cm on the right and 3 cm on the left (Figure 2). Nuclear medicine scan demonstrated evidence of persistent renal compromise with 65% and 35% function remaining in the right and left kidneys, respectively. Ureteral stricture excision and reimplantation were performed by the urology service. Histopathologic examination of excised ureteral segments demonstrated fibrosis and chronic inflammation without evidence of carcinoma or active tuberculous disease. The patient continues to follow with the urology service and is doing well.

## 3. Comment

Recent case reports of acute obstructive uropathy in pregnancy describe ureteral obstruction caused by either a severely retroflexed, incarcerated uterus [4] or an overdistended gravid uterus such as that encountered in multifetal gestations and polyhydramnios [5]. On review of the literature, most cases of genitourinary TB in pregnancy were published over forty years ago, leading practitioners to believe that genitourinary TB is more a historical interest than a contemporaneous consideration. In fact, genitourinary TB is estimated to represent up to 30% of extrapulmonary TB in some reports [2, 6] and is estimated to be the culprit in up to 8% of female infertility cases [7]. Although a 30% incidence is higher than the 5% overall rate reported in 2012 by the Centers for Disease Control (CDC), genitourinary TB is likely underdiagnosed given the insidious onset of symptoms. Screening for TB in high-risk populations is imperative in order to improve overall detection rates and heighten the practitioner's awareness of the possibility of genitourinary TB. The most common urinary symptoms reported include dysuria (34–56%), irritative voiding symptoms such as frequency and urgency (5–88%), abdominal pain (5–33%), and hematuria (12–45%) [7]. In the pregnant patient these symptoms may be misdiagnosed as acute cystitis, and recurrence of symptoms despite treatment should prompt further diagnostic evaluation. If evidence of obstructive uropathy is found, the practitioner must consider the diagnosis of genitourinary TB even in those patients of average or low risk.

Tuberculous mycobacteria spread hematogenously after primary pulmonary infection and can seed the renal cortex, adrenal glands, ureters, bladder, and reproductive organs [7]. This occurs in up to 20% of patients with active pulmonary TB and is usually asymptomatic initially [7]. Chronic inflammation and fibrosis lead to ureteral strictures in 10–56% of patients with genitourinary TB, and one-third will have bladder involvement [7]. Additionally, secondary urinary bacterial infections may be present in up to 50% of patients [7], as a result of chronic reflux and urinary stasis caused by irregular strictures and segmental ureteral dilation [6]. Given the potential morbidity of urinary tract infection during pregnancy and the number of patients with occult genitourinary involvement during active pulmonary disease, there may be a benefit to screening women with pulmonary TB for asymptomatic bacteriuria or initiating suppressive antimicrobial therapy. To our knowledge, this has not been evaluated or reported in the literature.

The findings of sterile pyuria and persistent hematuria with negative urine culture are classic findings of genitourinary TB [2]. The gold standard for diagnosis of genitourinary TB is demonstration of mycobacteria on at least one of usually three to six consecutive early morning urine specimens sent for Ziehl-Neelsen acid-fast stain and urine culture [7]. Acid-fast smears are most sensitive in the presence of active TB and may be negative if tuberculin shedding is intermittent or when there are fewer than 5,000 organisms per milliliter of sample [7]. Urine PCR for* M. tuberculosis *yields results within 48 hours and has a high reported sensitivity and specificity of 87–100% and 92–98%, respectively, using culture as the standard [7]. Because our patient had received 4 months of RIE therapy, there were likely few to no remaining tuberculous bacilli in her urine specimens to be detected. In this case, the diagnosis was one of exclusion and circumstance. In similar cases where clinical suspicion is high despite negative urine samples for tuberculous bacilli, a positive interferon gamma release assay such as QuantiFERON Gold or T-SPOT TB test might indirectly lead to a diagnosis, although these are not specific for genitourinary TB. Additionally, renal biopsy for tissue culture has the greatest yield but is invasive [7], and although not contraindicated in pregnancy, the need for anesthesia and potential morbidity limits its use.

The current recommended treatment regimen for genitourinary TB is identical to that for pulmonary TB. CDC guidelines state that pregnant women with TB should be treated with isoniazid, rifampin, and ethambutol for nine months [1]. No randomized trials have investigated the optimal duration of therapy for genitourinary TB, although the European Association of Urology recommends two months of RIPE therapy, followed by rifampin plus isoniazid for an additional four months [3]. Interestingly, spontaneous conception during RIE treatment, while reportedly rare [7], may lend credence to a possible link between our patient's two years of infertility and her TB infection. In addition to medical therapy, percutaneous urinary diversion is therapeutic and protective of further renal deterioration if recognized early in cases of complete obstruction [6]. Nephrectomy is reserved for a nonfunctioning kidney or renal disease causing hypertension [3]. With irreversible ureteral disease, as in our patient's case, definitive management includes surgical reimplantation if ureteral dilatation is unsuccessful [3].

In conclusion, genitourinary TB should be considered as a possible etiology of obstructive uropathy causing renal failure during pregnancy, especially in foreign-born and immunocompromised persons. Early recognition and prompt treatment can prevent further deterioration of renal function and optimize pregnancy outcomes.
